# SIGIRR participates in negative regulation of LPS response and tolerance in human bladder epithelial cells

**DOI:** 10.1186/s12865-015-0137-5

**Published:** 2015-12-03

**Authors:** Dan Li, Xin Zhang, Baiyi Chen

**Affiliations:** Department of Infectious Diseases, the First Affiliated Hospital of China Medical University, Shenyang, 110001 Liaoning China

**Keywords:** Urinary tract infection, Human bladder epithelial cells, Toll like receptor, Single immunoglobulin IL-1R-related receptor, Lipopolysaccharide, Endotoxin tolerance

## Abstract

**Background:**

The innate immune response of urinary tract is critically important in the defense to microbial attack. Toll-like receptor 4 (TLR4) controls initial mucosal response to uropathogenic *Escherichia coli* (UPEC). However, excessive and dysfunctional TLR signaling may result in severe inflammation and inappropriate tissue damage. Previous studies have demonstrated that single immunoglobulin IL-1R-related receptor/Toll IL-1 receptor 8 (SIGIRR/TIR8) is a member of the toll-interleukin-1 receptor (TIR) family that can negatively modulate TLR4 mediated signaling, but its role in the innate immunity of urinary tract infection remains incompletely defined. In this study, we investigated its cellular distribution and mechanisms involved within the human bladder epithelial cells after LPS stimulation.

**Results:**

Immunostaining, reverse transcription PCR and Western blot results showed that SIGIRR was constitutively expressed in the human bladder epithelial cell lines and was downregulated after LPS stimulation. To further define the role of SIGIRR, cells were transiently transfected with SIGIRR siRNA and stimulated with LPS. SIGIRR gene silencing augmented chemokine expression in response to LPS, as indicated by increased levels of IL-6 and IL-8 secretions in the supernatants compared with negative control siRNA. Furthermore, LPS tolerance, a protective mechanism against second LPS stimulation, was significantly reduced in SIGIRR siRNA transfected cells. Moreover, transient gene silencing augmented LPS-induced NF-κB and MAPK activation.

**Conclusions:**

In conclusion, our results suggest that SIGIRR plays an important role in the negative regulation of LPS response and tolerance in human bladder epithelial cells, possibly through its impact on TLR-mediated signaling.

## Background

Urinary tract infections (UTIs) are among the most common bacterial infections in humans and account for significant morbidity and mortality. About 50 % of women will experience at least one UTI in their lifetime, and about 25 % of them will suffer from one or more recurrent infections [1]. UTIs are caused by a number of bacterial pathogens and uropathogenic *Escherichia coli* (UPEC) remains the predominant uropathogen isolated in community-acquired uncomplicated infections (80 %) and hospital-acquired (50 %) infections [2–5]. Although our knowledge about pathogenesis of UTIs has advanced greatly in recent years, the precise mechanisms of specific host-pathogen interaction are not well understood.

Bladder epithelial cells (BECs) act as the first line of defense against ascending pathogens. BECs can recognize conserved pathogen-associated molecular patterns (PAMPs) via several kinds of pattern recognition receptors (PRRs), including Toll-like receptors (TLRs) which can control the innate host defense at mucosal surfaces and protect the mucosal barrier against bacterial attack. Several Toll-like receptors (TLRs) have been identified in bladder epithelial cells including TLR4, which recognizes lipopolysaccharide (LPS) from Gram-negative bacteria and plays a key role in inducing the inflammatory responses elicited by UPEC [6–11]. Upon stimulation by LPS, TLR4 initiates a signaling cascade involving myeloid differentiation factor 88 (MyD88), IL-1R associated kinases (IRAKs) and tumor necrosis factor receptor-associated factor 6 (TRAF6), leads to activation of nuclear factor kappa B (NF-κB) and mitogen-activated protein (MAP) kinases p38, JNK and ERK1/2 [12, 13]. Then transcriptions of various cytokines were stimulated including IL-6 and IL-8, two of the major cytokines that are constitutively produced by urinary epithelial cells following the bacterial infection [14, 15].

Although the TLR-mediated inflammatory response is critical for host defense against pathogenic bacteria, excessive and dysfunctional TLR signaling may result in severe inflammation and inappropriate tissue damage. Therefore, the intensity and duration of TLR responses must be tightly controlled. In fact, a number of negative regulators of TLRs have been identified [16], including single immunoglobulin IL-1R-related receptor/Toll IL-1 receptor 8 (SIGIRR/TIR8) which is a member of TLR/IL-1R superfamily and has been reported to inhibit NF-κB and JNK activation following stimulation of TLR family members, including TLR4 [17, 18]. The inhibitory activity was associated with trapping of IRAK-1 and TRAF-6 [18, 19]. Overexpression of SIGIRR reduced TLR-mediated activation of NF-κB and attenuated the production of inflammatory cytokines in vitro. In SIGIRR-deficient mice, LPS induced inflammatory responses were also enhanced [18]. The high expression of SIGIRR in epithelial cells indicates that SIGIRR may serve mainly to dampen the immune response in cells that are continually exposed to microorganisms [18, 20–24]. Although SIGIRR was recently shown to regulate inflammation in a mouse model of UTI in tubular epithelial cells [25], the cellular distribution and mechanisms involved within the human bladder epithelial cells after LPS stimulation remain incompletely defined.

In the present study, we characterized SIGIRR expression and modulation in human bladder epithelial cells, and investigated the role of SIGIRR in regulating the immune responsiveness during inflammation induced by LPS. Our results suggest that SIGIRR is constitutively expressed in human bladder epithelial cell lines and is downregulated after LPS stimulation. Lack of SIGIRR results in increased production of proinflammatory cytokines, and SIGIRR gene silencing cells have strikingly impaired LPS tolerance, showing that SIGIRR negatively regulates TLR signaling in human bladder epithelial cells.

## Results

### Induction of IL-6 and IL-8 secretions by LPS in human bladder epithelial cell lines

**Fig. 1:**
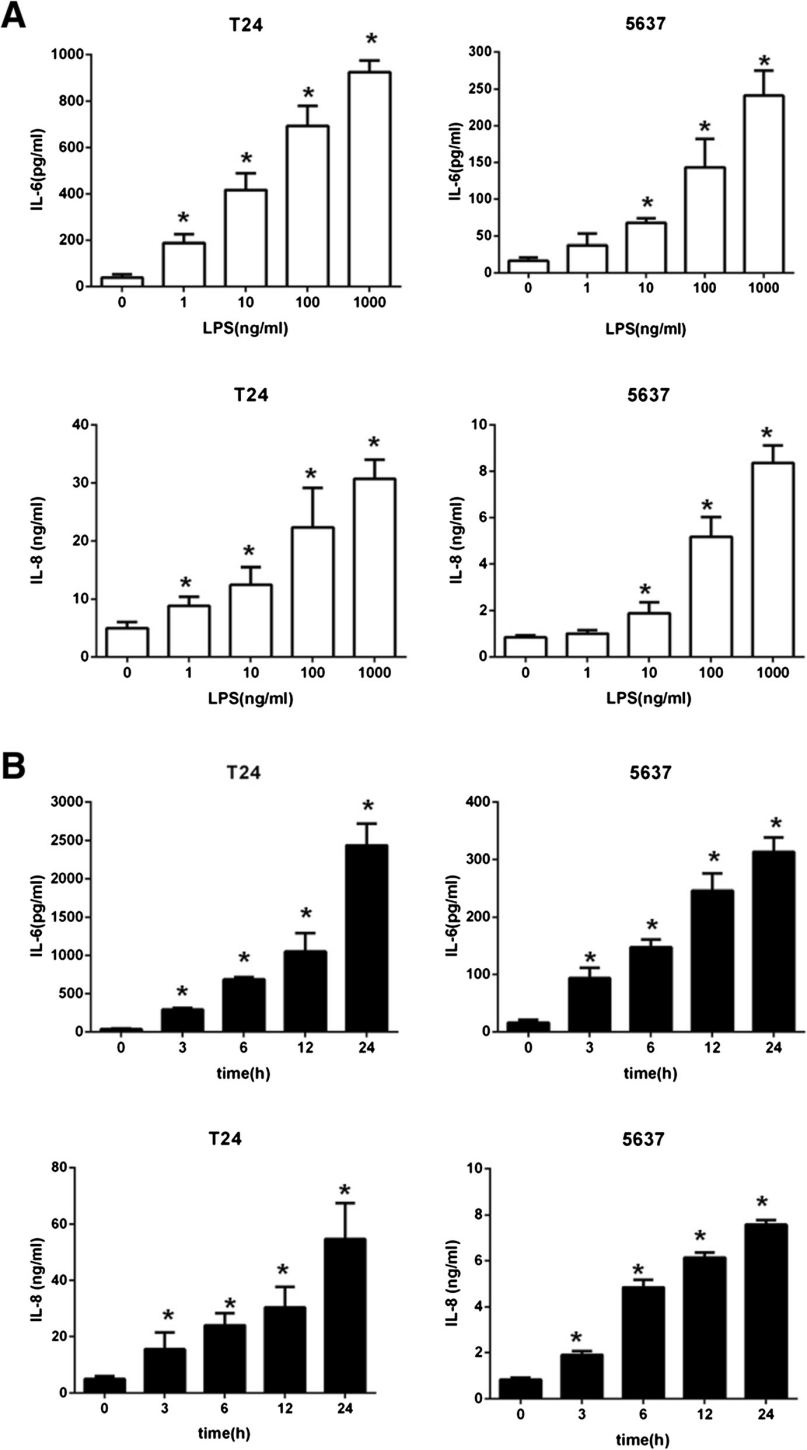
Induction of IL-6 and IL-8 in T24 and 5637 by LPS stimulation. **a** Cells were stimulated with log increments of LPS between 0 ng/ml and 1000 ng/ml, the supernatant levels of IL-6 and IL-8 were determined 6 h after incubation with LPS by ELISA, *p < 0.05 versus 0 ng/ml. **b** Cells were stimulated with 100 ng/ml LPS for 24 h, the supernatant levels of IL-6 and IL-8 measured by ELISA at the indicated times, *p < 0.05 versus 0 h. Experiments were performed at least three times in triplicate. Data represent the means ± SEM

The two human bladder epithelial cell lines were stimulated with LPS, and the levels of IL-6 and IL-8 in the culture supernatants were determined by ELISA. The time courses and dose dependency of IL-6 and IL-8 inductions are shown in Fig. 1. T24 and 5637 cells responded to LPS in a time and dose dependent manner, IL-6 and IL-8 levels were upregulated strongly in T24 cells and weakly in 5637 cells. Thus, the bladder epithelial cell lines appear to be uniformly LPS responsive, in contrast to renal epithelial cells, which are non-responsive to purified LPS [26]. As the two cell lines showed a sufficient response to ≥ 100 ng/ml LPS, this concentration was used in the remaining experiments.

### SIGIRR expression and alteration in human bladder epithelial cell lines

**Fig. 2:**
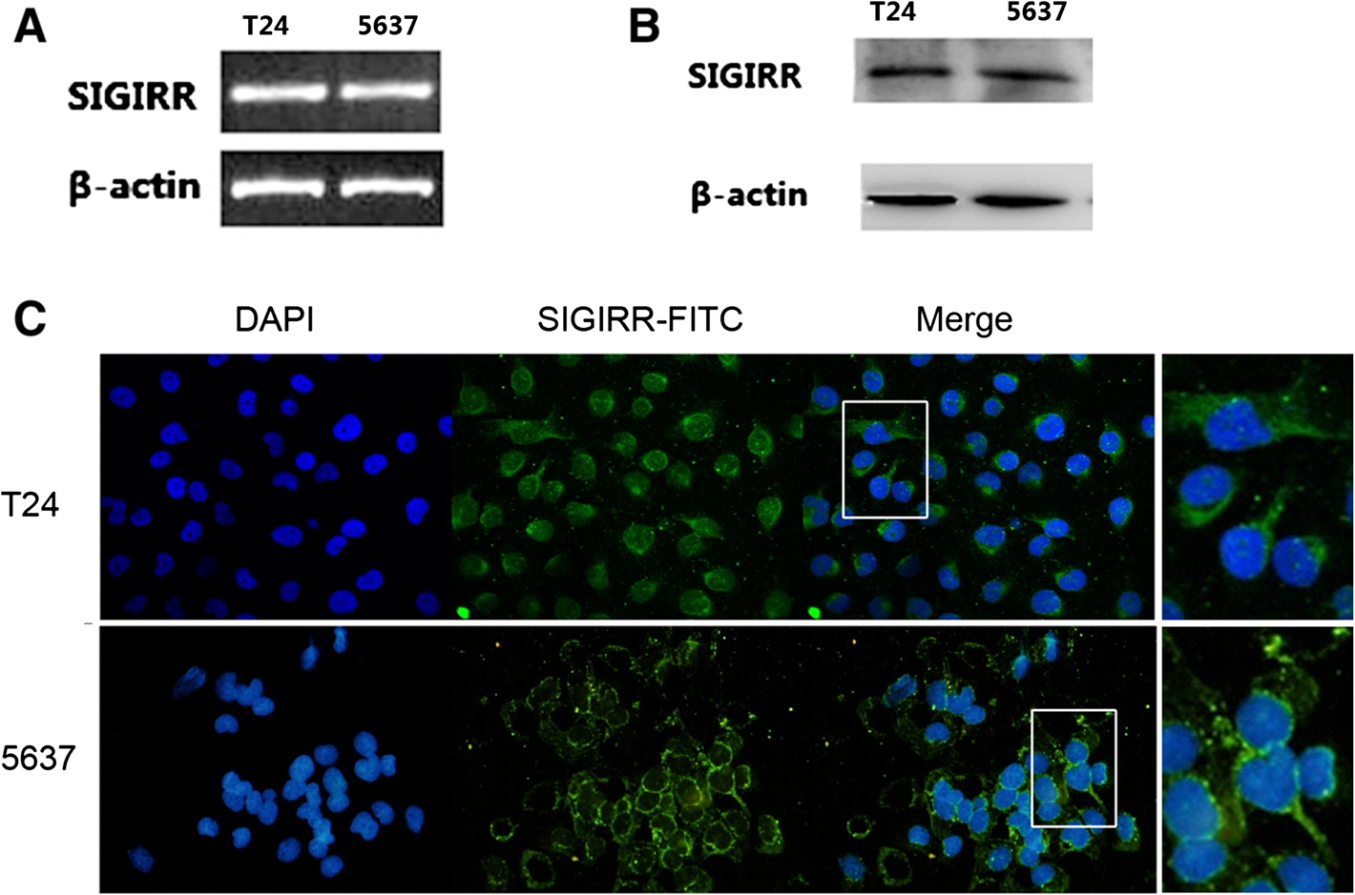
SIGIRR expression and localization in T24 and 5637 cells. SIGIRR mRNA expression (**a**) and protein expression (**b**) were detected by RT-PCR and Western blot in T24 and 5637 cells respectively. **c** Immunofluorescent stainning analysis showed that SIGIRR was localized in the cell nuclear membrane and cytoplasm

**Fig. 3:**
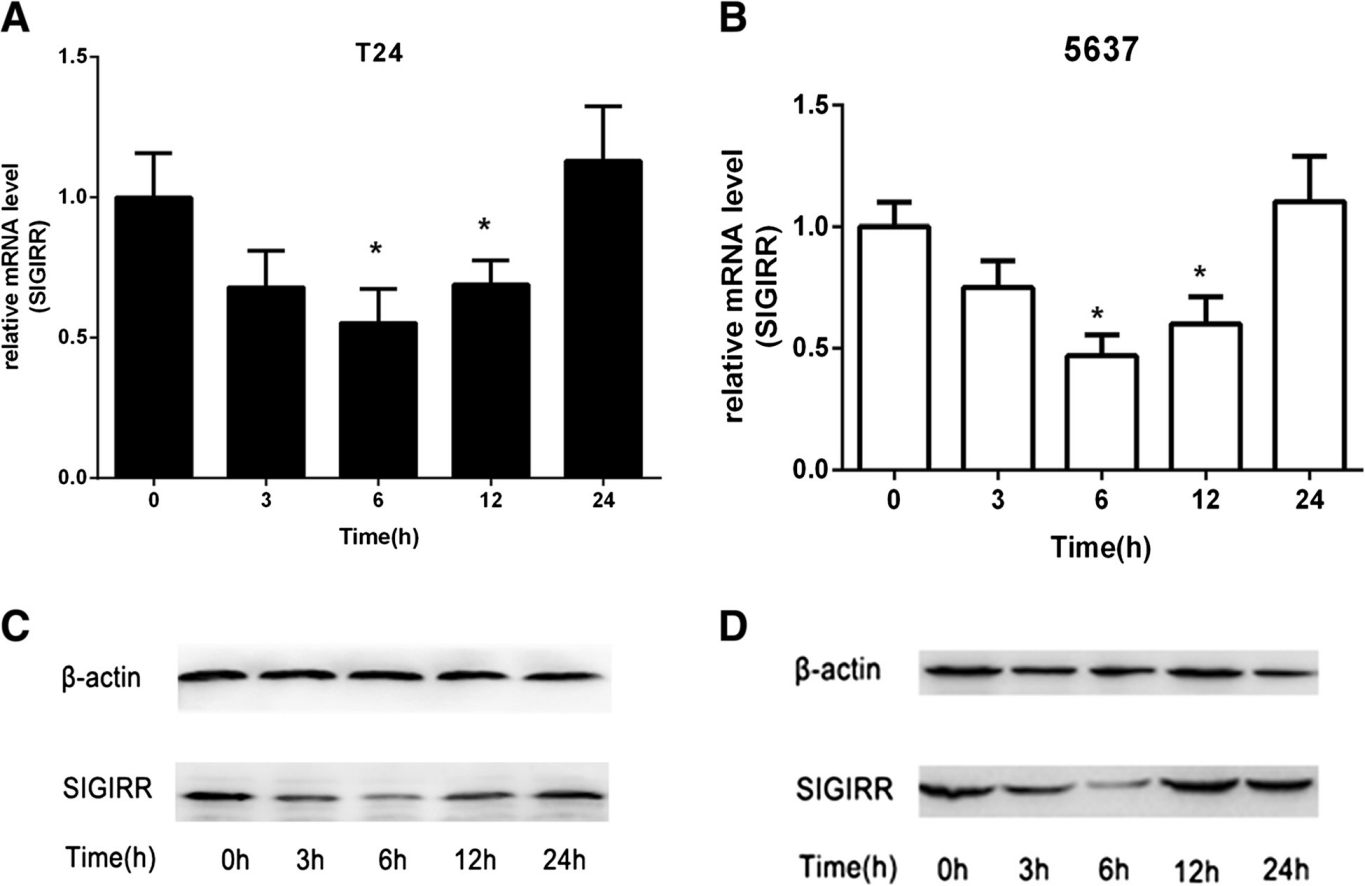
Downregulation of SIGIRR expression after LPS stimulation. **a** and **b** Changes in SIGIRR mRNA expression in T24 and 5637 cells after LPS stimulation at the indicated times were analyzed by real-time PCR. Data represent the means ± SEM from three independent experiments, *p < 0.05 versus 0 h. **c** and **d** Changes in SIGIRR protein expression in T24 and 5637 cells were analyzed by Western blot

To determine the presence of SIGIRR in T24 and 5637 cells and its modulation after LPS stimulation, we first tested SIGIRR mRNA and protein expression levels in the two cell lines using RT-PCR and Western blot respectively. The results suggest that SIGIRR is constitutively expressed (mRNA and protein) in nonstimulated cells (Fig. 2a and b). In addition, immunostaining analysis was performed for the detection of SIGIRR expression in human bladder epithelial cells. Confocal fluorescence images showed the localization of SIGIRR in the cell nuclear membrane and cytoplasm (Fig. 2c). To verify the influence of LPS stimulation on SIGIRR expression, we incubated the cells with 100 ng/ml LPS for indicated time courses. According to the results, SIGIRR mRNA was significantly downregulated after incubation with LPS for 6–12 h, and started to return to baseline levels after 24 h (Fig. 3a and b). Similar results were observed for protein levels (Fig. 3c and d).

### SIGIRR gene silencing augmented inflammatory response to LPS

**Fig. 4:**
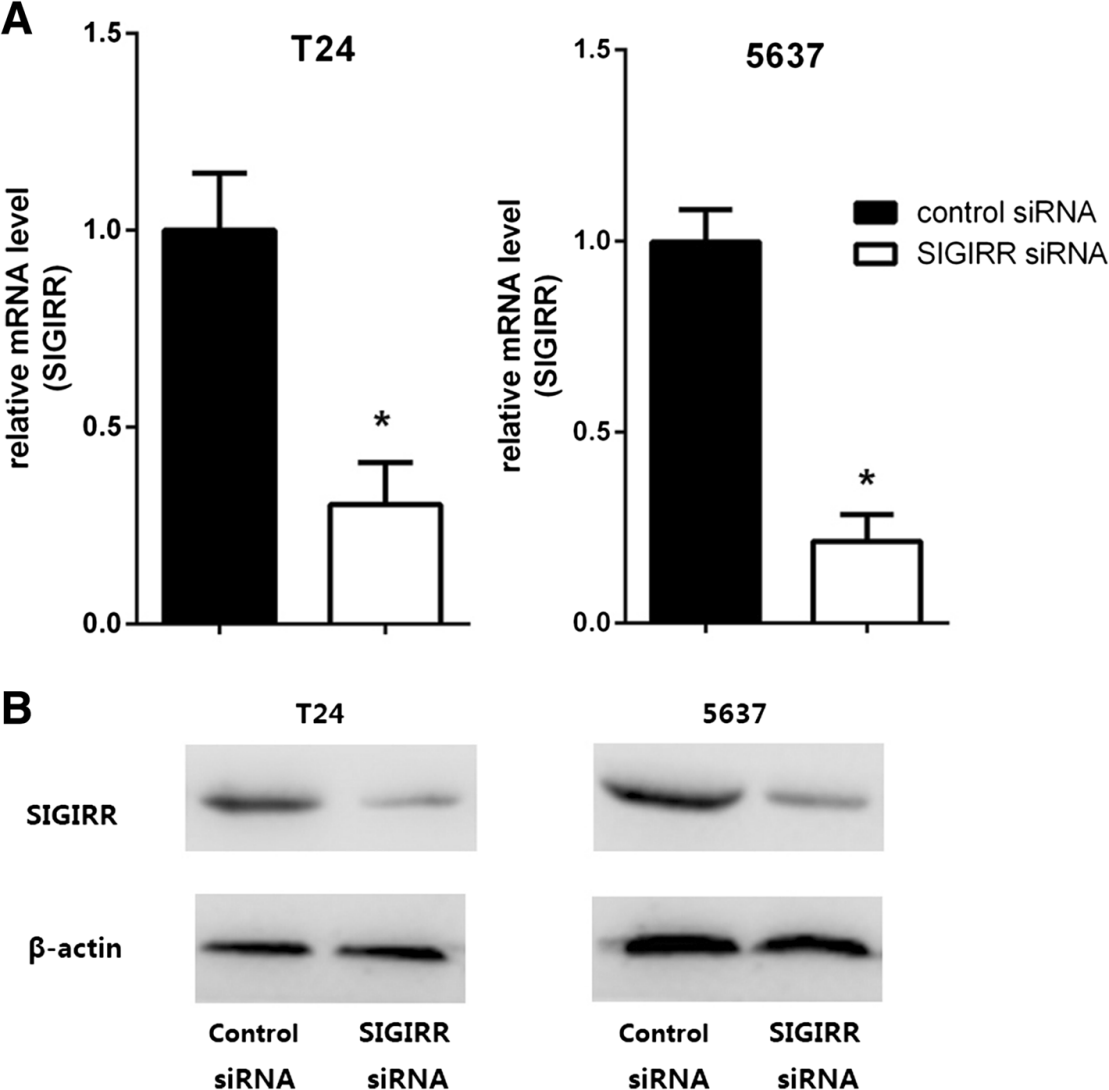
Transient gene silencing reduces SIGIRR expression in T24 and 5637 cells. Cells were transfected with SIGIRR and negative control siRNA in transfection reagents. Efficiencies of SIGIRR siRNA for target gene expression knock-down in cells were evaluated by real-time PCR (**a**) and Western blot (**b**). The mRNA levels of SIGIRR expression in each of the samples were normalized by GAPDH and compared to that of the negative control siRNA, *p < 0.05 versus negative control siRNA

**Fig. 5:**
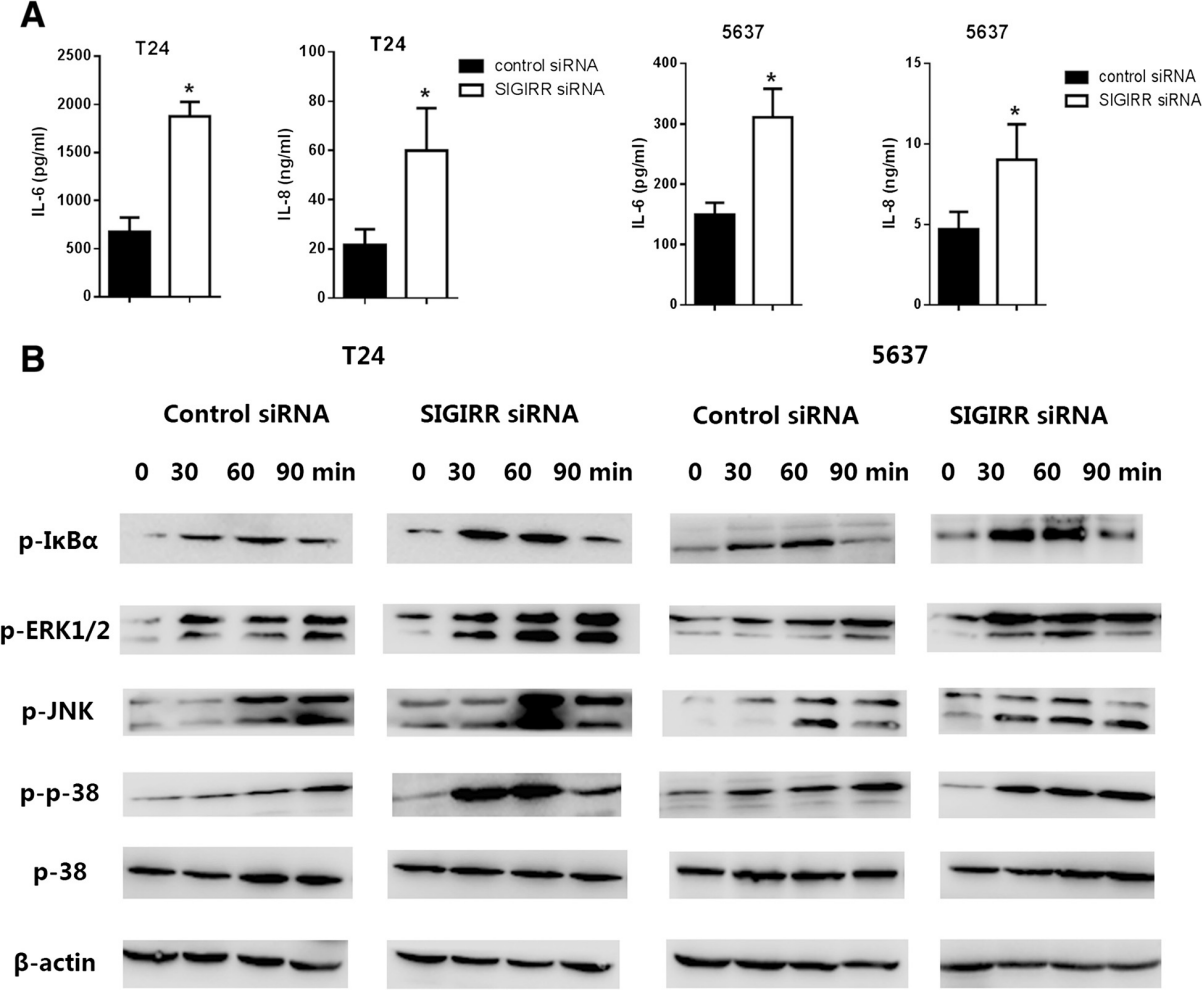
SIGIRR gene silencing augmented inflammatory response and signaling upon LPS stimulation. **a** Increased response in SIGIRR gene silencing cells upon LPS stimulation. SIGIRR and negative control siRNA transfected cells were exposed to 100 ng/ml LPS, cell culture supernatants were collected after 6 h, levels of IL-6 and IL-8 were quantified by ELISA. Data represent the means ± SEM from three independent experiments, *p < 0.05 versus negative control siRNA. **b** Increased signaling in SIGIRR gene silencing cells upon LPS stimulation. SIGIRR and negative control siRNA transfected cells were stimulated with 100 ng/ml LPS for the indicated periods. Cells lysates were blotted with the indicated antibodies

Previous studies demonstrate that SIGIRR deficiency leads to augmentation of TLR signaling. To clarify whether SIGIRR functions as a negative regulator in LPS stimulated bladder epithelial cells, we used a gene knockdown method with siRNA targeting SIGIRR. Cells were transfected with SIGIRR siRNA or negative control siRNA, then the efficiency of target gene knock-down was evaluated, which showed inhibition of SIGIRR expression by approximately 70–80 % in T24 and 5637 cells compared to the negative control siRNA (Fig. 4a and b). The effect of SIGIRR knockdown was maximal at 48–72 h post-transfection, and the reminder gene silencing studies were conducted within these time points. After confirming siRNA efficiency, the transfected cell lines were treated with or without LPS for 6 h, after which the IL-6 and IL-8 productions in culture supernatants were examined by ELISA. LPS mediated IL-6 and IL-8 productions in the targeted siRNA-treated cells were significantly greater than that in the negative control siRNA-treated cells (Fig. 5a), indicating that SIGIRR expressed in T24 and 5637 cells functions as a negative regulator of TLR-4 signaling.

### Enhanced TLR signaling in SIGIRR siRNA transfected cells

TLR stimulation activates NF-κB, JNK, p38 and ERK1/2 through the signaling molecules MyD88 and IRAK. We therefore examined the activation of these downstream effectors of TLR signaling in SIGIRR siRNA transfected cells. SIGIRR siRNA transfected cells were stimulated with LPS, and the activation of NF-κB, JNK, p38 and ERK1/2 were analyzed. Phosphorylations of JNK, p38 and ERK1/2 were faster and stronger in SIGIRR siRNA transfected cells than negative control siRNA transfected cells, indicating enhanced signaling in LPS-stimulated SIGIRR siRNA transfected cells and suggesting that SIGIRR negatively regulates these signaling pathways (Fig. 5b).

### Involvement of SIGIRR in LPS induced tolerance

**Fig. 6:**
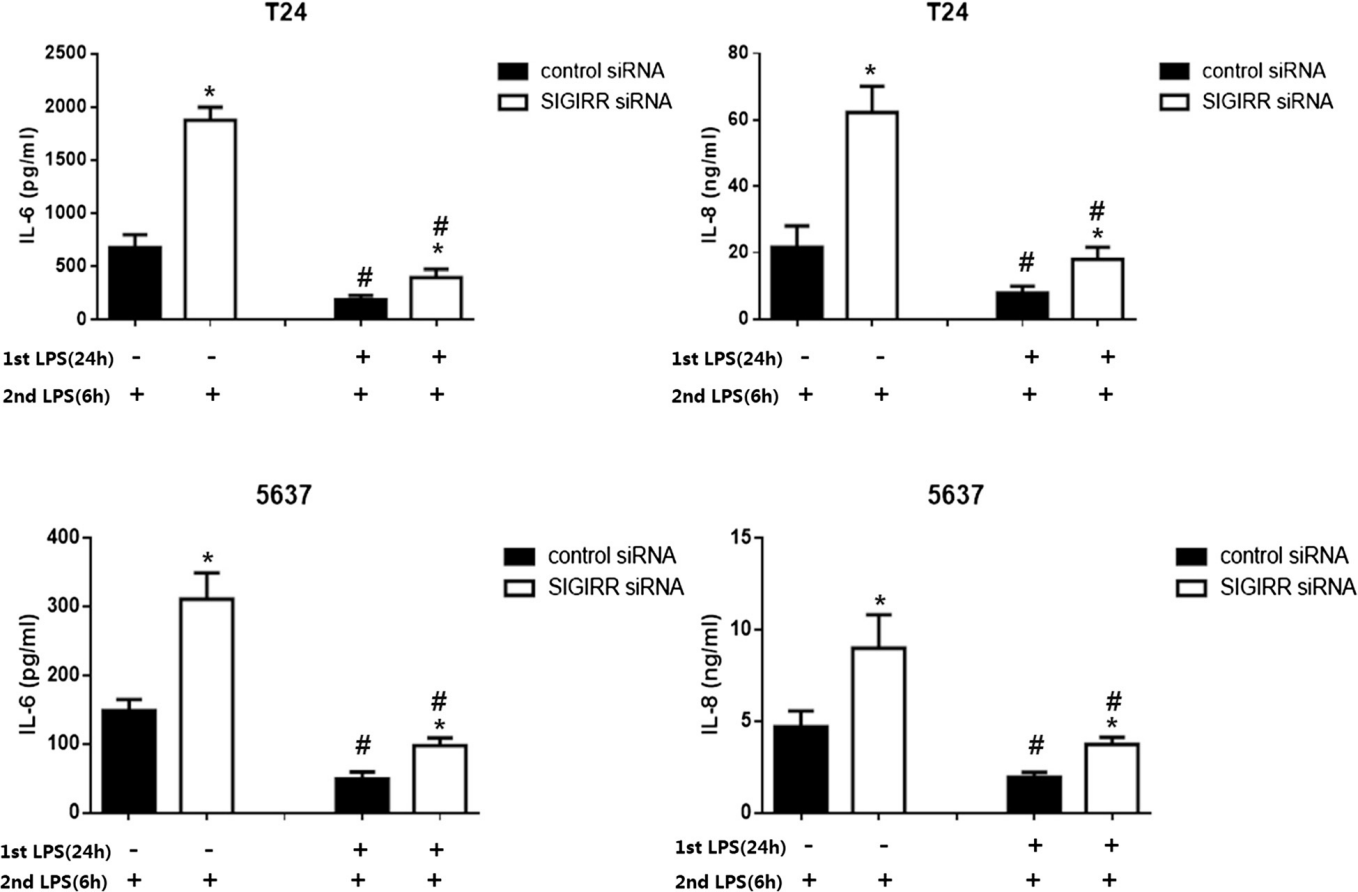
SIGIRR is required for LPS tolerance in T24 and 5637 cells. LPS tolerance was induced in SIGIRR and negative control siRNA cells by pretreatment with 100 ng/ml LPS (1st LPS) for 24 h or maintained in medium. After 24 h, the cells were washed with fresh medium and restimulated with 100 ng/ml LPS (2nd LPS) for 6 h. The levels of IL-6 and IL-8 in the supernatants were measured by ELISA. Experiment were repeated at least three times in triplicate with similar results, #*p* < 0.05 with respect to the same treatment but without pretreatment with LPS, *p < 0.05 versus negative control siRNA with the same treatment

Our results showed that SIGIRR is a negative regulator of TLR signaling which suggested that SIGIRR might be involved in the induction of LPS tolerance. To examine this, SIGIRR and negative control siRNA transfected cells were first stimulated with 100 ng/ml of LPS (primary LPS stimulation) or medium. After incubation for the indicated period (24 h), cells were restimulated with 100 ng/ml LPS (second LPS stimulation) for 6 h, the IL-6 and IL-8 concentrations in the supernatants were measured by ELISA. The levels of IL-6 and IL-8 were significantly reduced in cultures that had been pretreated with LPS for 24 h (Fig. 6). These data demonstrated that LPS tolerance can be induced in human bladder epithelial cells by pretreatment of the cells with LPS. However, SIGIRR siRNA transfected cells showed an impaired LPS tolerance, as the cytokine levels produced upon LPS restimulation in SIGIRR siRNA transfected cells were significantly higher than that in restimulated negative control transfected cells (Fig. 6).

## Discussion

In the present study, we examined the expression of SIGIRR in human bladder epithelial cells (BECs) and its alteration during inflammation. Our results suggested that SIGIRR gene silencing caused an increased response to LPS, in terms of exaggerated production of proinflammatory cytokines which was associated with enhanced TLR signaling and impaired tolerance to LPS. These are the first known results to shown the role of SIGIRR in human bladder epithelial cells during inflammatory response induced by LPS.

Single immunoglobulin IL-1-related receptor, also known as Toll IL-1 receptor 8 (SIGIRR/TIR8), is one of the transmembrane negative regulators of TLR signaling. SIGIRR is constituted by a single Ig extracellular domain with several N- and O-glycosylation sites, a transmembrane domain, an intracellular conserved TIR domain and a 95 amino acid-long tail at the C-terminal [17, 27]. Although SIGIRR has highly conserved TIR domain, it does not contain two amino acids (Ser447 and Tyr 536, replaced by Cys222 and Leu305), which were essential for the IL-1R signaling. The tissue expression of SIGIRR is ubiquitous, in particularly in kidney, digestive tract, liver, lung and lymphoid organs [17, 28], its cell-type expression is more specific, with extremely high expression in epithelial cells lines [18]. Thus it seems that SIGIRR regulates the immune response mainly at epithelial and mucosal sites. In this study, the expression of SIGIRR in T24 and 5637 cell lines, which are human bladder epithelial cell lines, were detected by immunostaining, RT-PCR and Western blot. We chose to use T24 and 5637 cells as they are similar to primary human bladder epithelial cells in that they express TLR4 and are sensitive to stimulation by LPS and *E.coli* [10, 14, 29, 30]. We found that SIGIRR was constitutively expressed in T24 and 5637 cells in mRNA and protein levels. Confocal fluorescence microscopy demonstrated that SIGIRR was expressed in the cell nuclear membrane and cytoplasm. These findings are consistent with earlier studies in which SIGIRR were stably overexpressed in human intestinal and airway epithelial cells [31, 32]. The location in human bladder epithelial cell lines suggests that SIGIRR may participate in the epithelial immune response during inflammation.

Previous studies found that murine and human SIGIRR expression were usually down-regulated by LPS stimulation or in other inflammatory conditions. For instance, SIGIRR expression was down-regulated at 6 and 12 h in mice after injection of a low dose of LPS in lung and kidney [18, 28]. A similar result showing the alteration of SIGIRR expression was also observed in the lung upon *Pseudomonas aeruginosa* infection or in the intestinal epithelial cells during inflammation in human ulcerative colitis [22, 33]. Our findings of decreased expression of SIGIRR following LPS stimulation are in concordance with previous studies. Real-time PCR analysis revealed that SIGIRR mRNA levels started to decrease at 3 h after exposure to LPS, reached the lowest level at about 6 h, and returned to baseline by 24 h after the cell recovered from LPS stimulation. Similar results were observed for protein levels. This was consistent with the findings in kidney that SIGIRR mRNA was significantly downregulated at early time points and started to increase toward baseline levels at later time point after UTI [34]. It seems that the downregulation is dependent upon decrease in binding of the transcription factor SP1 at the responsive element of the SIGIRR promoter. In the presence of LPS, the binding of SP1 to SIGIRR promoter consensus sites was reduced, resulting in transient inhibition of SIGIRR expression in epithelial cells [22]. It can be speculated that this downregulation would enhance an inflammatory response against urinary infection or elimination of microbes during tissue damage. While the subsequent upregulation could, in turn, ameliorate collateral damage due to an exaggerated or prolonged inflammatory response. However, LPS or cytokine exposure rather upregulates SIGIRR in monocytes in contrast with tubular epithelial cells, a finding consistent with the observation of SIGIRR expression in monocytes of patients with sepsis and cardiac arrest (RCA). This cell type-specific regulation may be associated with N- and O- glycosylation of SIGIRR in different cell origins [27, 35].

The pattern of SIGIRR consumption may indicate its functional involvement in ameliorating inflammation. Gene-targeted studies demonstrate that SIGIRR acts as a nonredundant negative regulator in dampening IL-1R and TLR-induced inflammation and tissue damage, including *tuberculosis*, *candidiasis*, *aspergillosis*, *Pseudomonas aeruginosa* infection and endotoxemia [18, 23, 33, 36, 37]. Next, in order to elucidate the role of SIGIRR in BECs innate immune response, we knocked-down SIGIRR expression by using gene silencing and found that inflammation was exacerbated by an elevated productions of IL-6 and IL-8 after the cells were stimulated with LPS. These findings indicate clearly that decreased expression of SIGIRR causes a significant enhancement of inflammatory response in BECs by dysregulating innate immunity. Previous mechanistic studies indicate that SIGIRR blocks TLR4 signaling by sequestering IL-1 receptor-associated kinase (IRAK) and tumor necrosis factor (TNF) receptor-associated (TRAF) 6, which are the key signaling proteins causing activation of NF-κB, JNK, p38, and ERK1/2 [19]. Therefore, the exacerbated response of SIGIRR siRNA transfected cells is likely the result of enhanced TLR signaling. Consistent with this, SIGIRR siRNA transfected BECs stimulated with LPS displayed increased NF-κB and MAP kinase activation, both well-characterized outputs of TLR stimulation. Our findings demonstrate that SIGIRR is an important negative regulator of LPS-induced BECs activation. Similar function was observed in modulation of adaptive immunity by effectively inhibiting IL-33 induced ERK, JNK, p38 and IκB phosphorylation and fine-tuning type1/type2 inflammation response [33, 38].

A prior exposure to bacterial products such as lipopolysaccharide (LPS) may induce a transient state of refractoriness to subsequent challenge that has been referred to as “tolerance”. Tolerance has been postulated as a protective mechanism limiting excessive inflammation and preventing septic shock [39]. The molecular mechanism of endotoxin tolerance is complicated, multiple mechanisms are involved including the deficient recruitment of the adaptor MyD88 to TLR4, decreased IL-1 receptor-associated kinase (IRAK) 4-MyD88 association, deficient IRAK1 activation, upregulation of negative regulators such as IRAK-M, suppressor of cytokine signaling 1 (SOCS-1), ST2 and SHIP [40–46]. Generally speaking, “tolerance” has a much more specific meaning in immune cells like monocyte/macrophage. Since “LPS tolerance” has been mentioned in the previous investigation on the ability of bladder epithelial cells to become tolerant to LPS stimulation, we used this term in our study [14]. With its high expression in epithelial cells and negative role in TLR signaling, we suppose that SIGIRR may play a critical role in the maintenance of innate immune tolerance to microbes. We found that BECs which were prestimulated with LPS had significantly reduced response to LPS, the results demonstrated that LPS tolerance can be induced in uroepithelial cells, which was consistent with previous report [14]. Moreover, SIGIRR siRNA transfected cells were significantly impaired in the development of tolerance upon repeated stimulation with LPS, which suggested the involvement of SIGIRR in LPS tolerance. Our results indicate that SIGIRR may be an important modulator in controlling the homeostasis and innate immune response of uroepithelium to bacterial products. Future studies are required to dissect its role in the mechanism and crosstalk of endotoxin tolerance.

## Conclusions

Taken together, we have identified the expression of SIGIRR in human bladder epithelial cell lines and its alteration after LPS stimulation. The negative role of SIGIRR in Toll like receptor signaling and its involvement in LPS tolerance in human uroepithelial cells indicate that SIGIRR is an important feedback regulator of innate immunity and plays a critical role in maintenance of epithelium homeostasis.

## Methods

### Cell culture

Human bladder epithelial T24 and 5637 cell lines were obtained from the Shanghai Institute of Cell Biology, Chinese Academy of Sciences. Cells were maintained in RPMI1640 medium (Gibco BRL, Gaithersburg, MD, USA) supplemented with 10 % fetal bovine serum (Hyclone, Logan, Utah), 2 mM L-glutamine, 100 IU/ml penicillin and 100 μg/ml streptomycin at 37 °C in a humidified atmosphere of 95 % air and 5 % CO2.

### Immunofluorescent staining

Immunofluorescent analyses of SIGIRR expression in T24 and 5637 cells were performed as follows. Cells were fixed with 4 % paraformaldehyde and then permeabilized with 0.2 % Triton X-100. After being blocked with 5 % BSA, cells were incubated with anti-SIGIRR antibody (1:100, Santa Cruz, CA, USA) at 4 °C overnight. The next day, after being washed with PBS, cells were incubated with a FITC-conjugated anti-goat secondary antibody (1:200, Beyotime, China) for 2 h and then stained with DAPI (Beyotime, China). Cells were analyzed under a confocal microscope (Leica SP1 and Leica SP2 UV, Leica Microsystem).

### RNA extraction and reverse transcription-polymerase chain reaction

Total RNAs were isolated using Trizol reagent (Invitrogen Corp., Carlsbad, CA, USA), according to the manufacturer’s instructions. cDNA was generated using PrimeScript RT reagent kit (Takara Biotechnology Co., Dalian, China) according to the manufacturer’s protocol, which was then amplified by PCR using specific primers for SIGIRR (forward, 5′- GAG AGC CGG AGC AGC GAA GT -3′; reverse, 5′- CCT GTT GAG CAG AGG AGC GA-3′, 173 bp) and β-actin (forward, 5′- CTC CAT CCT GGC CTC GCT GT-3′; reverse, 5′-GCT GTC ACC TTC ACC GTT CC-3′, 268 bp). PCR conditions consisted of 26 cycles at 94 °C for 30s, 58 °C for 30s, and 72 °C for 45 s for denaturation, annealing, and extension, respectively, followed by a final extension at 72 °C for 4 min. The PCR products were analyzed by electrophoresis on 2 % agarose gel.

### Real-time quantitative polymerase chain reaction

The expression levels of SIGIRR gene were analyzed by real-time quantitative PCR (Corbett Rotor-Gene), cells were harvested after LPS stimulation at the indicated times and siRNA transfection. Total RNAs were prepared using Trizol Reagent (Invitrogen, Carlsbad, CA) and cDNA was synthesized using the PrimeScript RT reagent kit (Takara Biotechnology Co., Dalian, China) according to the manufacturer’s protocol. Relative levels of SIGIRR gene expression were assessed using the SYBR Premix Ex Taq kit (Takara). Primers for real-time PCR were as follows: SIGIRR, forward 5′- GAG AGC CGG AGC AGC GAA GT -3′, reverse 5′- CCT GTT GAG CAG AGG AGC GA -3′; glyceraldehyde 3-phosphate dehydrogenase (GAPDH), forward 5′- CCA CAT CGC TCA GAC ACC AT-3′, reverse 5′- TGA CCA GGC GCC CAA TA-3′. Real-time PCR products were analyzed by ΔΔCt method and all mRNA expression levels were normalized to glyceraldehyde-3-phosphate dehydrogenase (GAPDH) expression in the respective cDNA preparation. All experiments were repeated three times in triplicate.

### Western blot

Cells were lysed with RIPA buffer (Sigma-Aldrich, St. Louis, MO) supplemented with fresh protease inhibitor phenylmethanesulfonyl fluoride on ice. Protein concentrations were measured using a BCA Protein Assay Kit (Pierce Biotechnology Inc., Rockford, IL, USA). Protein (50 μg) was boiled and loaded on 10 % sodium dodecyl sulfate polyacrylamide gel electrophoresis and transferred to a PVDF membrane (Millipore, Bioprocess Technology Center, Billerica, MA, USA). Membranes were blocked in 5 % non-fat milk and incubated overnight at 4 °C with the appropriate primary antibodies at dilutions specified by the manufacturer. Antibodies against SIGIRR, phospho-IκBα (p-IκBα), phospho-ERK1/2 (p-ERK1/2), phospho-JNK (p-JNK), phospho-p38 (p-p38), p-38 and β-actin were purchased from Santa Cruz Biotechnology (California, USA). They were next washed three times in 15 ml Tris-buffered saline with Tween 20 (TBS-T) and incubated with the corresponding horseradish peroxidase (HRP)-conjugated secondary antibodies (Zhongshan Golden Bridge Biotechnology, Beijing, China) at 1:1000 dilution in TBS-T for 2 h at room temperature with slightly shaking. Then the bound secondary antibodies were detected using ECL Western blotting Detection System (Amersham, Uppsala, Sweden).

### SIGIRR small interfering RNA transfection

Cells were seeded at the density of 2 × 10^5^ cells/well in six-well tissue culture plates. After incubation for 18–24 h, the cells (60–80 % confluence) were washed with PBS and transiently transfected with SIGIRR specific small interfering RNA (sc-61547; Santa Cruz Biotechnology, CA, USA) or negative control siRNA (sc-37007; Santa Cruz Biotechnology) mixed with transfection reagent (sc-29528; Santa Cruz Biotechnology) according to the manufacturer’s instructions. Fluorescein conjugated control siRNA (sc-36869) was used to monitor transfection efficiency. Transfection medium (sc-36868) were also purchased from Santa Cruz Biotechnology. At 48 and 72 h after transfection, real-time PCR and Western blot were used to determine the effectiveness of SIGIRR siRNA in suppression of mRNA and protein levels.

### Stimulation of human bladder epithelial cells and induction of LPS tolerance

T24 and 5637 cells were seeded at the density of 2 × 10^5^ cells/well in six-well tissue culture plates and grown to confluence over 2 days. The cells were treated with culture medium alone or medium containing 1 ng/ml, 10 ng/ml, 100 ng/ml or 1000 ng/ml of LPS (*Escherichia coli* serotype O55:B5, Sigma-Aldrich, St. Louis, MO). After incubation for 6 h, IL-6 and IL-8 levels in the supernatants were determined by ELISA. Then the nonstimulated cells were incubated with 100 ng/ml LPS for 24 h, culture supernatants were taken at 0, 3, 6, 12, 24 h and assayed for IL-6 and IL-8 concentrations by ELISA.

LPS tolerance was induced by pretreatment with 100 ng/ml LPS (1^st^ LPS). After 24 h, the supernatants were removed and the cells were washed once with fresh medium and restimulated with 100 ng/ml of LPS (2^nd^ LPS) for 6 h. The concentrations of IL6 and IL-8 in the supernatants were evaluated by ELISA and compared with that received the second stimulation alone.

### Determination of IL-6 and IL-8 by ELISA

IL-6 and IL-8 concentrations in the culture supernatants were measured by commercial ELISA kits (R&D Systems, Minneapolis, MN) according to the manufacturer’s instructions. All samples were assayed in triplicate.

### Statistical analysis

The data are presented as mean ± SEM. All statistical results were analyzed with Student’s *t*-test using GraphPad Prism 5.0 (GraphPad Software, San Diego, CA). Differences were considered significant at the level of *p* < 0.05.

## Abbreviations

SIGIRR/TIR8: single immunoglobulin IL-1R-related receptor/Toll IL-1 receptor 8
TLR: toll like receptor
LPS: lipopolysaccharide
UTI: urinary tract infection
UPEC: uropathogenic *Escherichia coli*
BEC: bladder epithelial cell
